# DNA damage-induced AIM2 pyroptosis in high glucose-induced proximal tubular epithelial cell

**DOI:** 10.3389/fcell.2024.1457369

**Published:** 2024-11-26

**Authors:** Lu’an Li, Li Zhang, Yating Cai, Jiaying Li, Siqi Zheng, Weiteng Wang, Yinwen Chen, Jieyi Luo, Ruizhao Li, Xinling Liang

**Affiliations:** ^1^ School of Medicine, South China University of Technology, Guangzhou, China; ^2^ Department of Nephrology, Guangdong Provincial People’s Hospital (Guangdong Academy of Medical Sciences), Southern Medical University, Guangzhou, China; ^3^ Guangdong Cardiovascular Institute, Guangdong Provincial People’s Hospital (Guangdong Academy of Medical Sciences), Guangzhou, China; ^4^ Ganzhou Hospital of Guangdong Provincial People’s Hospital, Ganzhou Municipal Hospital, Ganzhou, China

**Keywords:** AIM2, pyroptosis, diabetic nephropathy, DNA damage, high glucose

## Abstract

Pyroptosis is one of the ways to cause proximal tubular epithelial cell death in diabetic nephropathy (DN), but the exact mechanism remains unclear. Absent in melanoma 2 (AIM2), a sensor for double-stranded DNA, creates an inflammasome that triggers the cleavage of gasdermin D (GSDMD), leading to a type of inflammatory cell death called pyroptosis. This study investigated the role of AIM2 in pyroptosis within proximal tubular epithelial cells in DN. We observed significantly elevated AIM2 expression in renal tubules from DN patients and db/db mice, as well as in high glucose (HG)-induced Human Kidney-2 (HK2) cells. Besides, increased AIM2 expression was accompanied by activation of the pyroptosis pathway (cleaved-caspase-1, GSDMD-FL, GSDMD-NT) in the renal cortex of db/db mice and HG-induced HK2 cells *in vitro*. Knocking down GSDMD can reduce HG-induced HK2 cell death, indicating that HG triggers pyroptosis in HK2 cells. Furthermore, HG-induced pyroptosis was mitigated in HK2 cells with AIM2 knockdown using siRNA. Additionally, reducing ROS levels using NAC was able to attenuate HG-induced HK2 cells DNA damage, AIM2 activation, and pyroptosis. Notably, AIM2 upregulation was observed in renal biopsies from DN patients, with expression levels positively correlating with serum creatinine and inversely with estimated glomerular filtration rate (eGFR). Collectively, DNA damage caused by HG could result in the activation of the AIM2 inflammasome, leading to the pyroptosis of proximal tubular epithelial cells, indicating that targeting AIM2 could be a potential novel approach for treating DN.

## 1 Introduction

Diabetic nephropathy (DN) is a prevalent and severe chronic complication of diabetes. Approximately 30%–40% of patients with end-stage renal disease (ESRD) have developed DN (Zelnick et al., 2017; Umanath and Lewis, 2018). Despite the increasing standardisation of prevention and treatment strategies for DN, the incidence of DN is increasing annually, posing a significant global public health challenge. Historically, investigations into the pathophysiology of diabetic nephropathy have predominantly focused on glomerulopathy. Recent studies have shown that targeting tubulointerstitial abnormalities can alleviate the decline in renal function in DN (Mori et al., 2021). This highlights the importance of exploring the pathogenesis of renal tubular injury in DN.

Pyroptosis, a distinctive form of programmed cell death distinct from classical apoptosis, is an emerging focus in the investigation of renal tubular injury in DN (Li et al., 2024). Classical pyroptosis is initiated by the cleavage of gsdermin D (GSDMD) by activated caspase-1, resulting in the formation of an N-terminal peptide. This peptide induces pore formation and leads to cell rupture, ultimately inducing cell death (Lamkanfi and Dixit, 2014). The activation of caspase 1 depends on the creation of inflammasomes, which consist of the receptor, the adapter (ASC), and the downstream cysteine protease (caspase-1). The activation of receptors, including NLR family proteins, Absent In Melanoma 2 (AIM2), and Pyrin, facilitates the recruitment of ASC and caspase-1 to form inflammasomes, resulting in caspase-1 self-cleavage and activation (Yu et al., 2021). Emerging research on DN indicates elevated expression of pyroptosis marker proteins, GSDMD and caspase-1, in renal tubular cells of DN patients, with GSDMD expression positively correlated with tubular injury (Yuan et al., 2022). Consistent with this, *in vitro* high glucose (HG)-induced HK2 cell models and DN animal models has revealed increased levels of cleaved-caspase-1 and GSDMD-NT (Lan et al., 2022; Wen et al., 2022; Cui et al., 2023). The inhibition of caspase-1 has been shown to mitigate HG-induced cell death in HK2 cells and to ameliorate tubular injury in DN mice, implying that pyroptosis contributes to the pathogenesis of DN (Wen et al., 2022). However, the precise mechanism by which this occurs remains to be elucidated.

AIM2 is an inflammasome-forming DNA-sensing receptors (Rathinam et al., 2010). It is recognized for detecting pathogen-associated or host-derived cytoplasmic dsDNA, which triggers the recruitment of ASC and caspase-1 to form caspase-dependent inflammasomes, thereby promoting pyroptosis (Fernandes-Alnemri et al., 2009; Hornung et al., 2009). Recent research has indicated that AIM2 can also initiate pyroptosis by sensing nuclear DNA damage (Hu et al., 2016; Lammert et al., 2020). The overproduction of reactive oxygen species (ROS) is a key factor in DNA damage (Srinivas et al., 2019). ROS are implicated in the pathogenesis of various diseases, including cancer, diabetes, and heart disease, and are also essential in the development of DN (Giacco and Brownlee, 2010). However, whether ROS activate AIM2 through DNA damage in DN remains to be further explored. This study aimed to determine whether AIM2 regulates renal proximal tubular pyroptosis in DN and whether the activation of AIM2 is mediated by ROS-induced DNA damage.

## 2 Materials and methods

### 2.1 Human renal biopsy samples

DN renal biopsy samples were acquired from the Department of Nephrology, Guangdong Provincial People’s Hospital. Normal control samples were obtained from patients with renal tumour resection who were excluded from diabetes mellitus, and all operations were performed by the Department of Urology, Guangdong Provincial People’s Hospital. The clinical data shown in Table 1 were collected for all samples. All studies were conducted in accordance with the tenets of the Declaration of Helsinki and were approved by the Research Ethics Committee of Guangdong Provincial People’s Hospital after obtaining informed consent from the subjects (Ethics No. KY2024-199-02).

**TABLE 1:** The clinical parameters of patients performed with immunofluorescence.

Patients ID	Gender	Age	Weight (Kg)	Pathology	uPCR (mg/g Cr)	uACR (mg/g Cr)	Scr (μmol/L)	eGFR (mL/min/1.73m^2^)
1	Female	25	69	Diabetic Nephropathy	19,065.1	8589.92	104.28	64.28
2	Male	48	62.8	Diabetic Nephropathy	1565.38	868.37	156.30	44.60
3	Female	54	68.1	Diabetic Nephropathy	2971.98	1641.42	108.96	49.60
4	Male	45	72.3	Diabetic Nephropathy	2011.66	1420.35	205.81	32.6
5	Male	39	85.5	Diabetic Nephropathy	8414.59	4079.70	290.69	22.40
6	Male	58	65	Diabetic Nephropathy	6735.08	3541.41	295.7	19.2
7	Male	37	61.1	Diabetic Nephropathy	6345.46	3953.36	277.24	24.06
8	Male	71	67	Diabetic Nephropathy	7666.38	4264.96	258.88	25.88
9	Female	64	51.7	Diabetic Nephropathy	2547.73	1453.39	293.49	13.9
10	Male	51	57	Diabetic Nephropathy	7845.21	3324.08	735	6.7
11	Male	60	80.5	Diabetic Nephropathy	2084.65	1089.87	476.6	10.81
12	Male	54	72	Renal tumour patient	NA	NA	136.39	50.3
13	Female	48	56.5	Renal tumour patient	NA	NA	108	52.3
14	Female	64	55	Renal tumour patient	NA	NA	77.71	69.5
15	Male	48	66	Renal tumour patient	NA	NA	130.66	55.2
16	Male	79	55	Renal tumour patient	NA	NA	117.45	50.5
17	Male	42	71	Renal tumour patient	NA	NA	114.33	67.7

Scr, serum creatinine; uPCR, urine protein creatinine ratio; uACR, urinary albumin creatinine ratio; eGFR, estimated glomerular filtration rate.

### 2.2 DN animal model

Eight-week-old male db/db mice and age-matched wild-type (db/m) mice were purchased from the Nanjing Biomedical Research Institute of Nanjing University and housed at the Animal Centre of Guangzhou Forevergen Biosciences Co., Ltd., where they were maintained under relatively constant room temperature (25°C ± 2°C), humidity (55% ± 5%) and photoperiod (12/12 h) to simulate normal physiological environments, with free access to standard rodent chow and water, and maintained for 12 weeks. Blood, urine and kidney samples were then collected to determine blood glucose, Urine albumin-to-creatinine ratio (UACR) levels and renal pathological changes to confirm the success of the DN model. All animal care and experiments were conducted in accordance with the Guidelines for the Care and Use of Laboratory Animals of the Guangdong Provincial People’s Hospital and approved by the Research Ethics Committee of the Guangdong Provincial People’s Hospital (Ethics No. KY2024-199-02).

### 2.3 Urine analysis

The Mouse Albumin Kit (Bethyl Laboratories Inc., TX, United States) was utilized to measure mouse urinary albumin, while the Creatinine Kit (Cayman Chemical, MI, United States) was used to measure mouse urine creatinine. All procedures were performed according to the standard procedures of the kits.

### 2.4 Renal histologic analysis

Mouse kidneys were immersed in 4% paraformaldehyde and fixed for 24 h at 4°C. They were then embedded in paraffin and sectioned at 4 μm. Staining with HE, MASSON, and PAS was carried out as directed.

### 2.5 Cell culture and treatment

The Human Kidney-2(HK2) cells were acquired from the American Tissue Culture Collection (ATCC; Rockville, MD, United States) and cultured as previously described (Xie et al., 2021). The cells were subjected to the following interventions: (1) treatment with normal glucose (CON, 5.3 mmol/L), high glucose (HG, 30 mmol/L) and normal glucose plus mannitol (MA, 24.7 mmol/L, same as osmolality control) for 24, 48 and 72 h (2) exposure to NAC (5 mmol/L; Selleck, TX, United States), an inhibitor of ROS, for 1 h, followed by the substitution of the medium with HG for an additional 72 h.

### 2.6 siRNA transfection

siRNA sequences targeting human AIM2 and GSDMD were synthesised by RiboBio Co., Ltd. HK2 cells were transfected with siRNA at a concentration of 50 nmol/L for 6 h using Lipofectamine 2000 transfection reagent (Thermo Fisher Scientific, MA, United States), after which the medium was replaced with HG medium and incubated for an additional 72 h.

### 2.7 Western blot

Western blot was performed as previously described (Zhang et al., 2017; Xie et al., 2021). The antibodies used were anti-AIM2 (1:1000; cat. no. 20590-1-AP, Proteintech, Wuhan, China), anti-GSDMD (1:1000; cat. no. ER1901-37, Huabio, Hangzhou, China), anti-caspase1 (1:1000; cat. no. 3866S, Cell Signalling Technology, MA, United States), anti-γH2AX (1:1000; cat. no. 29380-1-AP, Proteintech, Wuhan, China) and anti-GAPDH (1:5000; cat. no. 60004-1-Ig, Proteintech, Wuhan, China).

### 2.8 Immunofluorescence

Immunofluorescence was performed as previously described (Zhang et al., 2017; Xie et al., 2021). The antibodies used were anti-AIM2 (1:200; cat. no. 20590-1-AP, Proteintech, Wuhan, China), anti-γH2AX (1:200; cat. no. 29380-1-AP, Proteintech, Wuhan, China) and anti-ASC (1:100; cat. no. ab175449, Abcam, MA, United States). The positive staining areas in each image of the kidney sections were analysed using ImageJ.

### 2.9 ROS detection

HK2 cells were incubated with DCFH (50 μmol/L; sigma, Germany) for 15 min at 37°C without light, then washed three times with PBS and imaged by confocal microscopy.

### 2.10 Flow cytometric analysis

The Annexin V-FITC/propidium iodide (PI) apoptosis kit (KeyGEN BioTECH, Jiangsu, China) was used to detect HK2 cell death. Briefly, HK2 cells were washed three times with ice-cold PBS and cells were digested with 0.25% EDTA-free trypsin (Gibco, MA, United States), cells were collected and washed twice with PBS and 500 μL of binding buffer was used to resuspend the cells. Then 5 μL each of FITC Annexin V and PI were added and incubated at room temperature for 15 min, followed by analysis of cell fluorescence using a BD FACSVerse™ flow cytometer.

### 2.11 Data analysis

Data were presented as mean ± standard deviation. Data were statistically analysed using SPSS (version 23.0) and results were plotted using GraphPad Prism 9. Differences between the two groups were analysed using Student’s t-test and two-tailed significance test. Multiple comparisons between groups were analysed by one-way ANOVA with Bonferroni’s adjusted/Tukey’s test or Dunnett’s T3 test. Correlations between the two variables were analysed using Spearman’s rho correlation coefficients. *p*-values less than 0.05 were considered statistically different.

## 3 Results

### 3.1 AIM2 is significantly elevated in proximal renal tubular epithelial cells of DN patients

**FIGURE 1:**
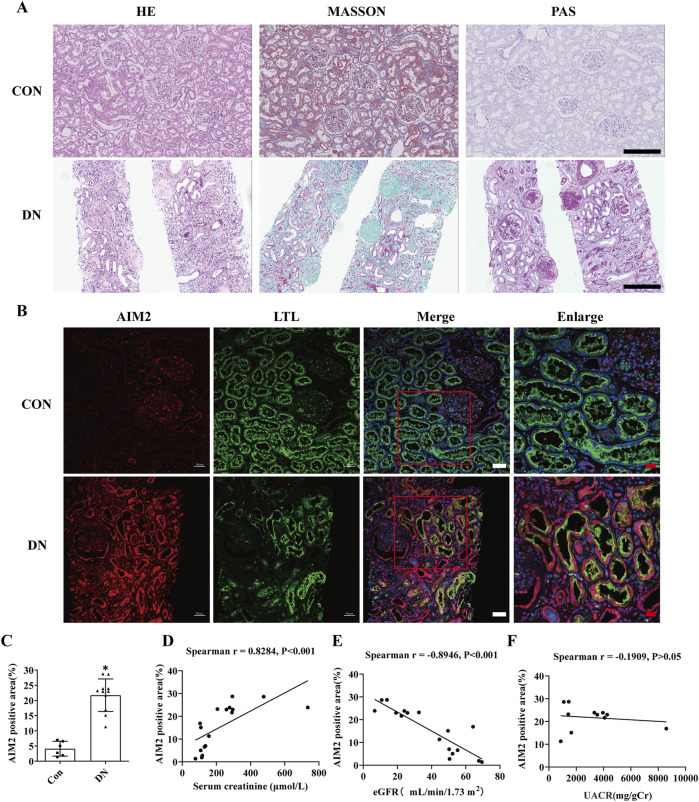
AIM2 is significantly elevated in proximal renal tubular epithelial cells of DN patients. **(A)** Representative pathological images in kidney sections from DN patients and normal kidneys. Scale bar: black 300 μm **(B)** Representative immunofluorescence images of AIM2 (red) and LTL (green) in kidney sections from DN patients and normal kidneys. Scale bar: white 50 μm, red 20 μm. **(C)** Quantitative fluorescence analysis of AIM2 in DN (n = 11) and normal (n = 6) kidney sections. **(D–F)** Correlation between renal AIM2 expression and serum creatinine (Scr, n = 17), estimated glomerular filtration rate (eGFR, n = 17) or urine albumin-to-creatinine ratio (UACR, n = 11) in all subjects. Data are shown as Mean ± SD. * vs. Con, *p* < 0.05.

We obtained renal tissue sections from 11 patients clinically diagnosed with DN by renal pathology (Figure 1A), and also obtained normal renal sections from six patients with tumors. AIM2 expression was assessed using immunofluorescence, revealing significantly elevated AIM2 fluorescence in various renal cell types, particularly in proximal tubular epithelial cells (identified with LTL), in DN patients as compared to the control group (Figures 1B, C). AIM2 was observed in both cytoplasm and nucleus (Figure 1B). We quantified the area of fluorescence-positive regions across all images and conducted correlation analysis with serum creatinine, estimated glomerular filtration rate (eGFR) and urine albumin-to-creatinine ratio (UACR). Strikingly, AIM2 levels in the kidney positively correlated with serum creatinine (Figure 1D), negatively with eGFR (Figure 1E) and showed no correlation with UACR (Figure 1F).

### 3.2 AIM2 is significantly elevated in proximal tubular epithelial cells of db/db mice

**FIGURE 2:**
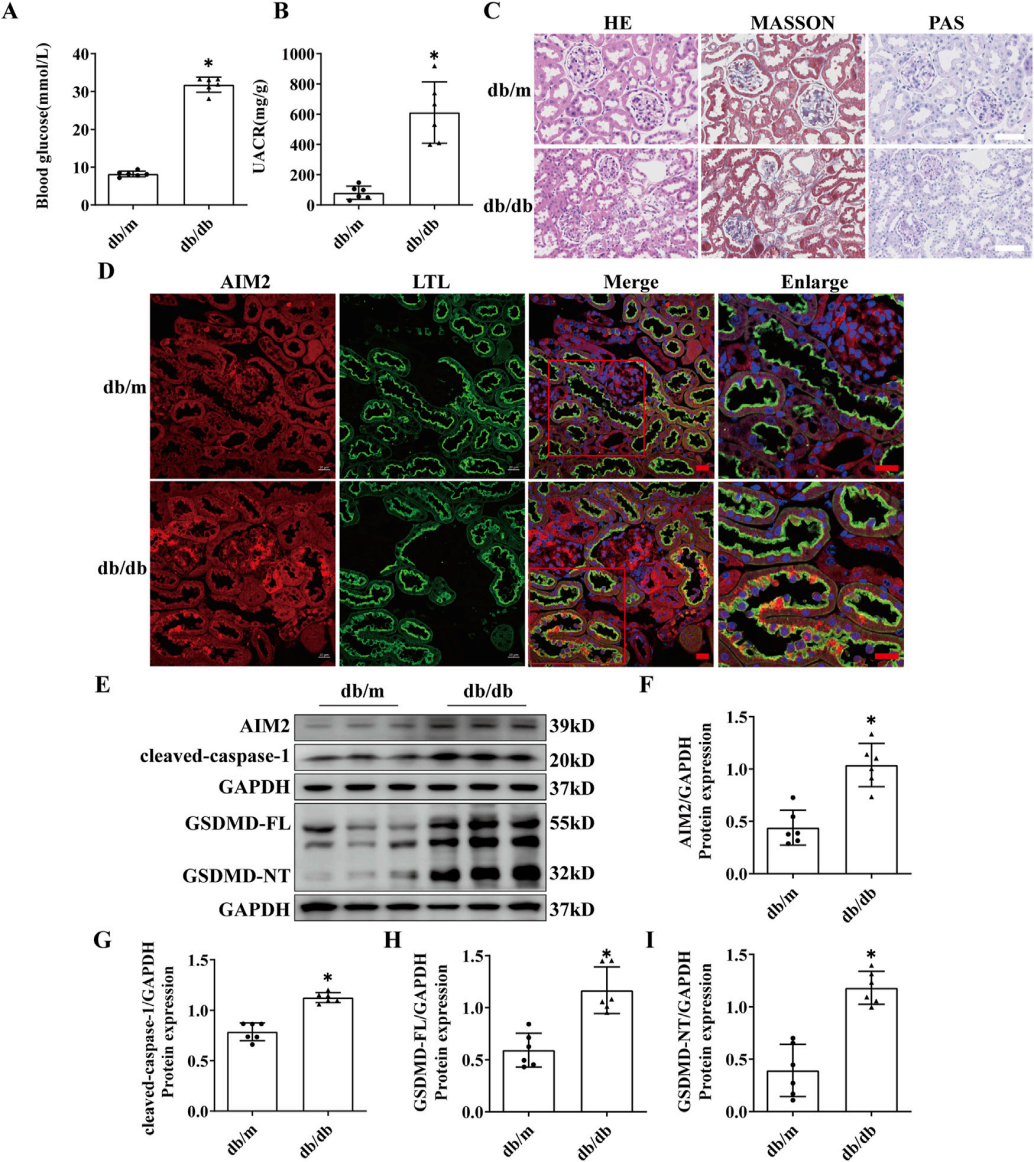
AIM2 is significantly elevated in proximal tubular epithelial cells of db/db mice. **(A)** Blood glucose in mice (n = 6 mice per group). **(B)** Mouse urine albumin-to-creatinine ratio (UACR, n = 6 mice per group). **(C)** Representative pathological images in kidney sections of db/m and db/db mice. Scale bar: white 50 μm. **(D)** Representative immunofluorescence images of AIM2 (red) and LTL (green) in kidney sections of db/m and db/db mice. scale bar: red 20 μm. **(E–I)** Representative Western blot images and quantification of AIM2 (n = 6 mice per group), cleaved-caspase1 (n = 6 mice per group), GSDMD-FL (n = 6 mice per group) and GSDMD-NT (n = 6 mice per group) expression in the renal cortex of db/m and db/db mice. Data are shown as Mean ± SD. * vs. db/m, *p* < 0.05.

Compared to db/m mice, db/db mice exhibited significantly elevated blood glucose and UACR levels at 20 weeks of age (Figures 2A, B). Histopathological staining revealed significant mesangial matrix expansion in the glomeruli of db/db mice, accompanied by mild tubular injury (Figure 2C). These findings collectively indicated the successful establishment of the DN model. Immunofluorescence revealed a notable increase in AIM2 expression within proximal renal tubular epithelial cells in db/db mice (Figure 2D), which corroborated our findings in human DN tissues. Western blot analysis further demonstrated that AIM2 expression was significantly increased in the renal cortex of db/db mice compared to db/m mice (Figures 2E, F).

AIM2, a canonical receptor involved in mediating pyroptosis, prompted us to investigate the protein expression within the pyroptosis pathway, including GSDMD-FL, GSDMD-NT, and cleaved-caspase-1. The expression of GSDMD-FL, GSDMD-NT and cleaved-caspase-1 was significantly increased in the renal cortex of db/db mice (Figures 2E, G–I), suggesting the presence of pyroptosis in the kidneys of DN mice and may be correlated with AIM2 expression.

### 3.3 HG induced the activation of AIM2 and the subsequent occurrence of pyroptosis in HK2 cells

**FIGURE 3:**
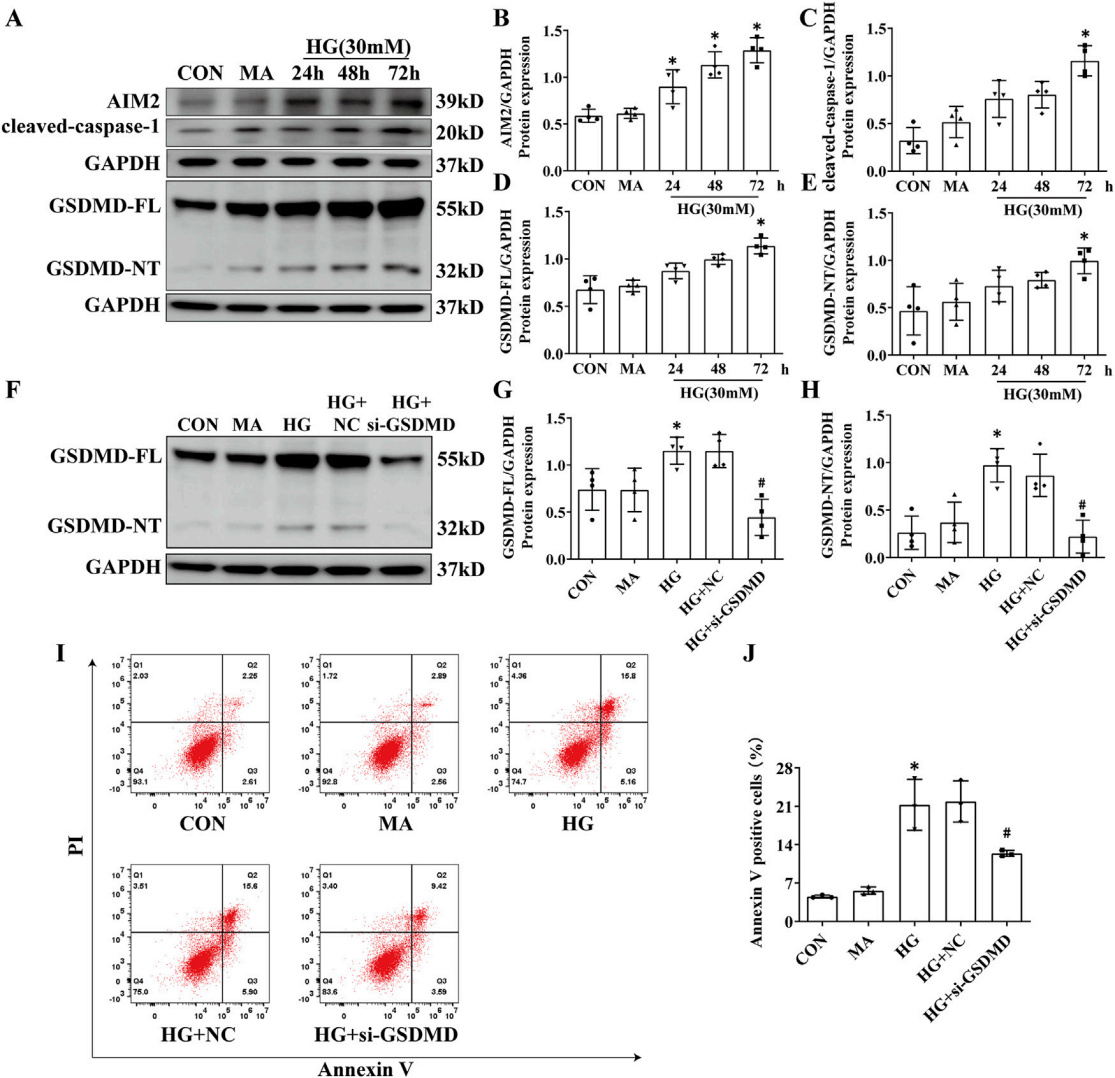
HG induced the activation of AIM2 and the subsequent occurrence of pyroptosis in HK2 cells. HK2 cells were treated with high glucose (HG) at a concentration of 30 mmol/L for 24, 48 and 72 h. Knockdown of the GSDMD in HK2 cells under HG intervention with a small interfering RNA. **(A)** Representative Western blot images of AIM2, cleaved-caspase1, GSDMD-FL and GSDMD-NT expression in HK2 cells. **(B–E)** Quantification of AIM2 (n = 4), cleaved-caspase1 (n = 4), GSDMD-FL (n = 4), GSDMD-NT (n = 4) expression in HK2 cells. **(F)** Representative Western blot images of GSDMD-FL and GSDMD-NT expression in HK2 cells. **(G–H)** Quantification of GSDMD-FL (n = 4), GSDMD-NT (n = 4) expression in HK2 cells. **(I–J)** Flow cytometry analysis to assess the regulation of HK2 cell death by knocking down GSDMD (n = 3). Data are shown as Mean ± SD. * vs. MA, # vs. HG + NC, *p* < 0.05.

We exposed HK2 cells to HG at a concentration of 30 mmol/L for 24, 48 and 72 h *in vitro*. Western blot analysis demonstrated a progressive increase in AIM2 expression with extended HG treatment, peaking at 72 h compared to the CON group (Figures 3A, B). Meanwhile, the expression of GSDMD-FL, GSDMD-NT and cleaved-caspase-1 were significantly elevated in HG-treated HK2 cells after 72 h (Figures 3A, C–E). Knocking down GSDMD can reduce HG-induced HK2 cell death (Figures 3F–J), indicating that HG triggers pyroptosis in HK2 cells. These findings imply a potential correlation between AIM2 and pyroptosis in HG-treated HK2 cells.

### 3.4 Knockdown of AIM2 diminishes HG-induced pyroptosis in HK2 cells

**FIGURE 4:**
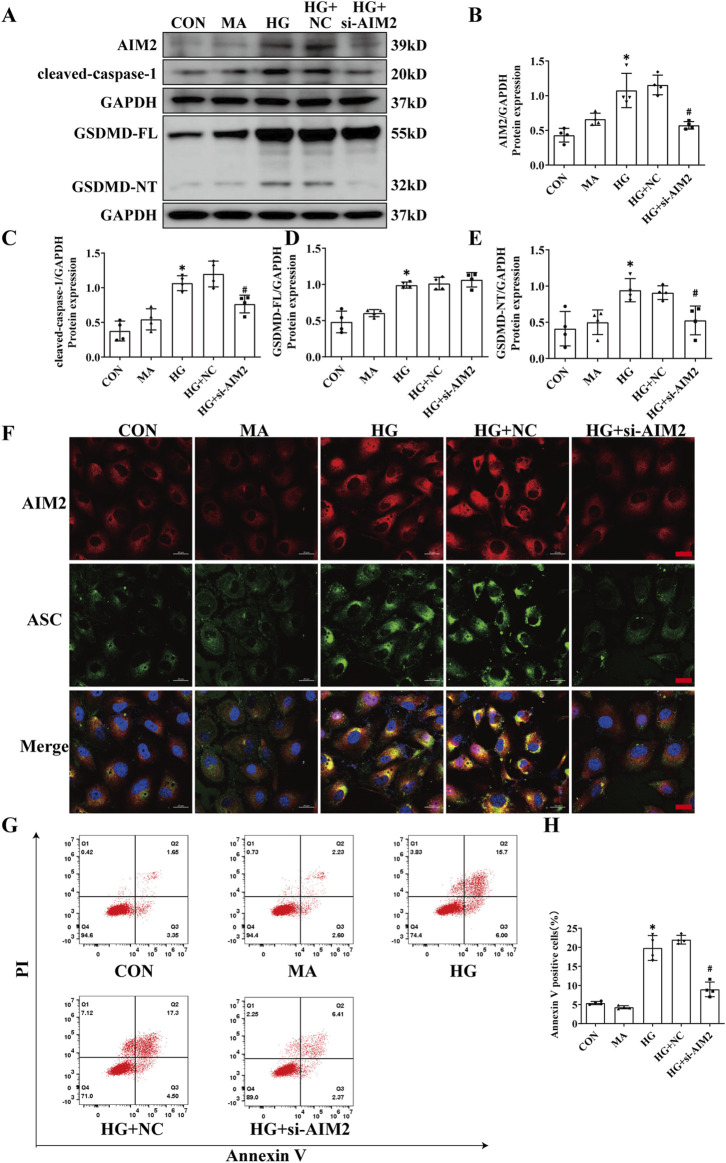
Knockdown of AIM2 diminishes HG-induced pyroptosis in HK2 cells. Knockdown of the AIM2 in HK2 cells under HG intervention with a small interfering RNA. **(A–E)** Representative Western blot images and quantification results of AIM2, cleaved-caspase1, GSDMD-FL, GSDMD-NT expression in HK2 cells (n = 4). **(F)** Representative cellular immunofluorescence images of AIM2 (red) and ASC (green) in HK2 cells. scale bar: red 20 μm. **(G–H)** Flow cytometry analysis to assess the regulation of HK2 cell death by knocking down AIM2 (n = 4). Data are shown as Mean ± SD. * vs. CON, # vs. HG + NC, *p* < 0.05.

To further explore the role of AIM2 in HG-induced pyroptosis in HK2 cells, we utilized AIM2-targeting siRNA to suppress AIM2 expression. Compared with the HG + negative control (NC) group, siRNA targeting AIM2 significantly reduced the protein expression of AIM2 (Figures 4A, B). Simultaneous knockdown of AIM2 markedly reduced the upregulation of cleaved-caspase-1 and GSDMD-NT induced by HG, while it did not affect the protein expression of GSDMD-FL (Figures 4A, C–E). Cellular immunofluorescence showed that AIM2 knockdown significantly decreased HG-induced ASC aggregation and expression (Figure 4F). Flow cytometry analysis demonstrated that AIM2 knockdown significantly lowered the population of annexin V-positive cells, indicative of reduced cell death, following HG treatment (Figures 4G, H). These findings suggest that AIM2 mediates pyroptosis in HG-exposed HK2 cells.

### 3.5 ROS inhibitor NAC diminishes AIM2 expression, DNA damage and attenuates pyroptosis in HK2 cells

**FIGURE 5:**
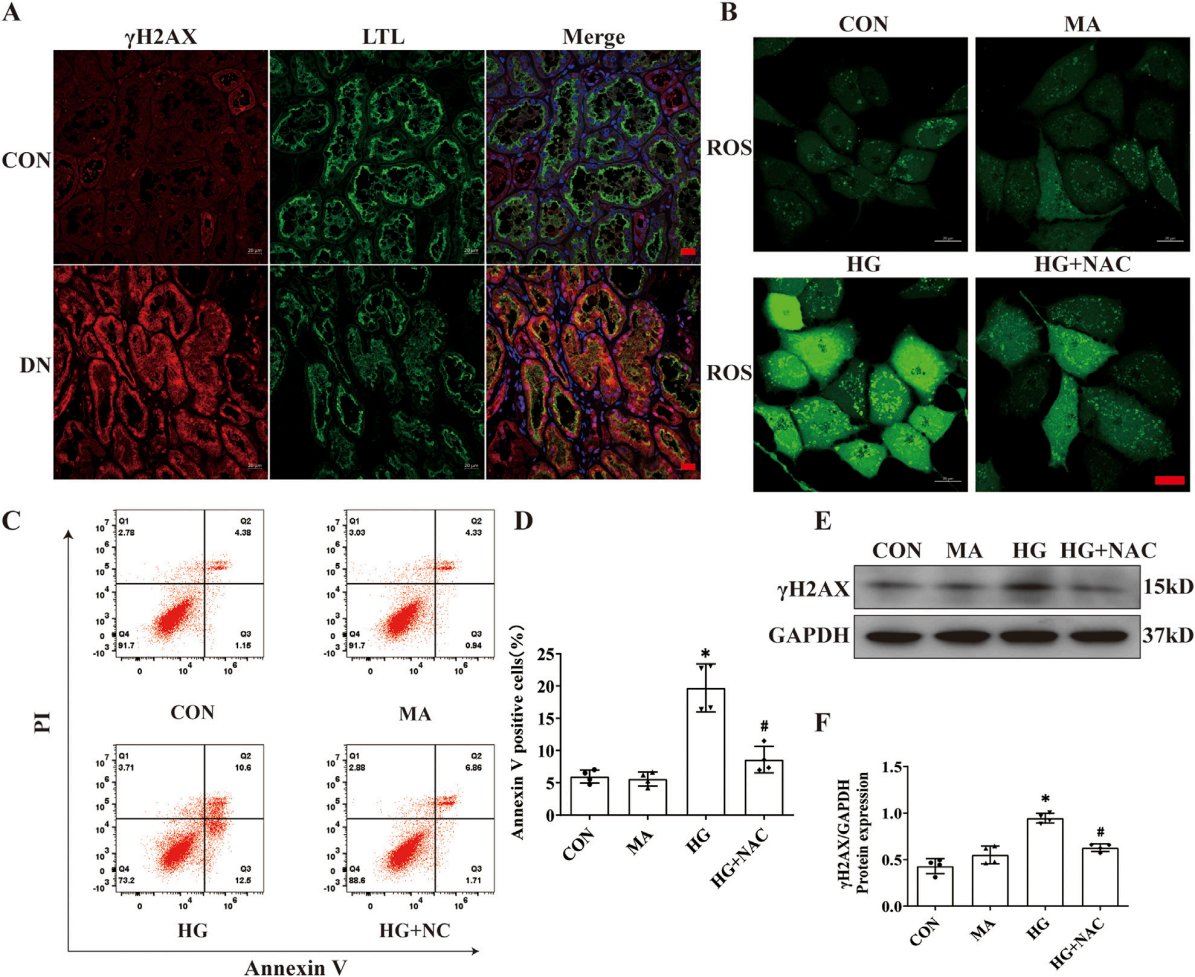
NAC diminishes DNA damage and HK2 cell death. Inhibition of ROS by NAC in HK2 cells with HG intervention. **(A)** Representative immunofluorescence images of γH2AX (red) and LTL (green) in kidney sections from DN patients and normal kidneys. Scale bar: red 20 μm. **(B)** Representative cellular immunofluorescence images of ROS in HK2 cells. scale bar: red 20 μm. **(C–D)** Flow cytometry analysis to assess the regulation of HK2 cell death by NAC (n = 4). **(E–F)** Representative Western blot images and quantification results of γH2AX (n = 4) expression in HK2 cells. Data are shown as Mean ± SD. * vs. CON, # vs. HG, *p* < 0.05.

**FIGURE 6:**
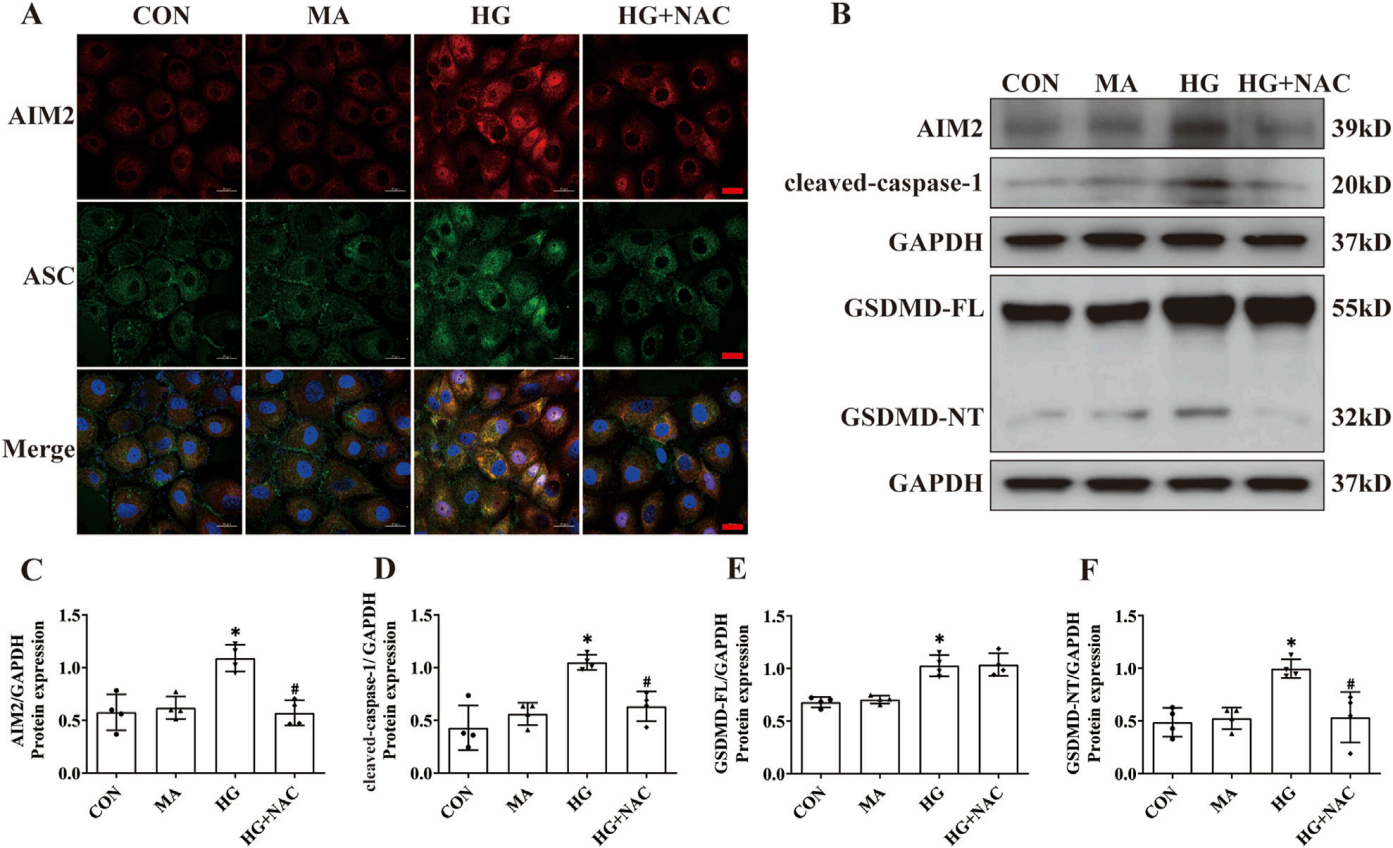
NAC diminishes expression of AIM2 and HG-induced pyroptosis in HK2 cells. **(A)** Representative cellular immunofluorescence images of AIM2 (red) and ASC (green) in HK2 cells. scale bar: red 20 μm. **(B–F)** Representative Western blot images and quantification results of AIM2, cleaved-caspase1, GSDMD-FL, GSDMD-NT expression in HK2 cells (n = 4). Data are shown as Mean ± SD. * vs. CON, # vs. HG, *p* < 0.05.

DNA damage, occurring in the nucleus, has been implicated in AIM2 activation (Hu et al., 2016). In this investigation, we observed an increase in the expression of the DNA damage marker γH2AX within the proximal renal tubular epithelial cells of DN patients (Figure 5A), indicating that DNA damage may be a pivotal factor in the activation of AIM2 in these cells. Given that ROS substantially contribute to DNA damage in DN (Holterman et al., 2015), we employed NAC, a ROS inhibitor (Pedre et al., 2021), to counteract HG-induced effects in HK2 cells. Compared to the HG group, NAC reduced the HG-induced increase in ROS levels (Figure 5B). Flow cytometry analyses revealed that NAC significantly attenuated the number of HG-induced annexin V-positive cells (Figures 5C, D) and decreased the protein expression of the DNA damage marker γH2AX (Figures 5E, F). Immunofluorescence results showed that NAC was able to reduce the nuclear translocation of AIM2 (Figure 6A), and WB showed that the protein expression of AIM2 was similarly reduced with NAC treatment (Figures 6B, C). This suggests that inhibition of ROS can mitigate DNA damage, consequently reducing AIM2 activation. In addition, NAC also diminished HG-induced ASC aggregation and expression (Figure 6A), as well as cleaved-caspase-1 and GSDMD-NT expression, without affecting GSDMD-FL protein expression (Figures 6B, D–F). These findings suggest that NAC attenuates HG-induced DNA damage in HK2 cells, thereby inhibiting AIM2-mediated pyroptosis.

## 4 Discussion

AIM2 is a pivotal receptor in the mediation of pyroptosis and plays a significant role in various kidney diseases. Our current research revealed that AIM2 expression was elevated in proximal renal tubular of DN patients, with increased expression positively correlating with creatinine levels and negatively with eGFR. Similarly, increased AIM2 expression was found in the proximal renal tubules of db/db mice and was associated with pyroptosis pathway. Notably, AIM2 knockdown reduced pyroptosis in HG-treated HK2 cells. Concurrently, we discovered that NAC, a ROS inhibitor, could suppress DNA damage and decrease the protein expression of AIM2, subsequently inhibiting pyroptosis.

Recent research has shown that pyroptosis is present in tubular cells in DN. In diabetic nephropathy (DN) patients, DN mouse models, and *in vitro* high glucose (HG)-induced HK2 cell models, increased expression of pyroptosis marker proteins, cleaved-caspase-1 and GSDMD-NT, has been observed in tubular cells (Lan et al., 2022; Wen et al., 2022; Yuan et al., 2022; Cui et al., 2023). Furthermore, both inhibition of caspase-1 and knockdown of GSDMD were found to mitigate HG-induced pyroptosis in HK2 cells *in vitro*, and caspase-1 inhibition alleviated tubular injury in DN mice (Wen et al., 2022; Yuan et al., 2022). Nevertheless, the mechanisms that trigger pyroptosis warrant further investigation. Recent evidence indicates that AIM2 is upregulated in the renal tubules of DN (Komada et al., 2018). AIM2, as a well-defined pyroptosis receptor, has been demonstrated to mitigate renal injury in ischaemia/reperfusion-induced AKI mice by inhibiting the AIM2 inflammasome (Yang et al., 2023), suggesting that AIM2 may mediate pyroptosis in renal proximal tubule during renal diseases. In our study, AIM2 expression was significantly elevated in both human and animal renal proximal tubules, with increased AIM2 expression positively correlating with blood creatinine levels and negatively with eGFR. Thus, we hypothesised that AIM2 may be involved in pyroptosis of renal proximal tubular epithelial cell in DN. HK2 cells have been reported to undergo pyroptosis after HG treatment *in vitro*, but the time of induction was inconsistent between different studies (Zhu et al., 2020; Cui et al., 2023; Lv et al., 2023). Our *in vitro* HG model revealed that the activation of GSDMD-NT, a key molecule in pyroptosis, peaked at 72 h, coinciding with increased AIM2 expression. Further studies showed that the expression of GSDMD-NT and the cell death rate of HK2 cells was significantly decreased after knockdown AIM2 in HK2 cells, suggesting that AIM2 is involved in HG-induced pyroptosis. The involvement of AIM2 in pyroptosis is caused by the activation of caspase1 via the induction of ASC polymerisation, resulting in the formation of GSDMD-NT peptide (Barnett et al., 2023). Consistently, our results also demonstrate that AIM2 knockdown decreases ASC aggregation and caspase-1 activation in HK2 cells.

Activation of the AIM2 inflammasome is usually due to AIM2 detecting pathogen-associated or host-derived cytoplasmic dsDNA. Research indicates that exogenous bacteria or viruses, such as Francisella tularensis and human papillomavirus (Reinholz et al., 2013; Man et al., 2015), are phagocytosed by macrophages and their dsDNA is released into the cytoplasm to activate the AIM2 inflammasome. In addition, certain viral infections or tumors cause cells to exhibit disruption of nuclear membrane integrity during the course of the disease and release dsDNA into the cytoplasm, leading to activation of AIM2 inflammasome (Di Micco et al., 2016). However, DN is a non-infectious microinflammatory disease, suggesting that there may be a different form of AIM2 inflammasome activation. AIM2 has been shown to sense DNA damage in the nucleus (Hu et al., 2016; Lammert et al., 2020; Li et al., 2021). When macrophages are exposed to ionizing radiation, AIM2 localizes to the nucleus, colocalizing with γH2AX and ASC, indicating its ability to detect DNA damage and initiate inflammasome assembly (Hu et al., 2016). Moreover, during neuronal development, AIM2 also senses DNA damage and induces pyroptosis, which has the effect of removing damaged neuronal cells (Lammert et al., 2020). Our study revealed that AIM2 translocates to the nucleus both *in vivo* and *in vitro*, suggesting that AIM2 may contribute to the activation of the inflammasome by sensing DNA damage. ROS are one of the major stimuli that cause DNA damage (Srinivas et al., 2019) and are an integral part of the pathogenesis of DN (Giacco and Brownlee, 2010). In this study, the increased protein expression of γH2AX (DNA damage marker protein) and AIM2, along with upregulated pyroptosis, were significantly reversed after treatment with the ROS inhibitor NAC under HG conditions, suggesting that the reduction of DNA damage may attenuates AIM2-mediated pyroptosis.

Furthermore, we found that AIM2 is expressed not only in the renal tubular epithelial cells of DN but also in a variety of renal cell types, including glomerular cells. Current research indicates the presence of pyroptosis in multiple cell types within the glomeruli in DN, including podocytes and endothelial cells (Cheng et al., 2021; Shen et al., 2024). However, the mechanisms triggering pyroptosis remain unclear. This implying that AIM2 may play a role in inducing pyroptosis in multiple cell types in DN, although further investigation is needed to elucidate this aspect. Collectively, we found that AIM2 expression was elevated in proximal renal tubular *in vivo* and vitro model of diabetic nephropathy. The increased expression of AIM2 positively correlated with serum creatinine levels and negatively with eGFR in DN patients. Importantly, we first demonstrated that AIM2 is an important contributor to increased pyroptosis in proximal tubular epithelial cell of DN. Pharmacological targeting AIM2 may provide a novel approach for the treatment of DN.

## Data Availability

The original contributions presented in the study are included in the article/Supplementary Material, further inquiries can be directed to the corresponding authors.
